# Advances in Linear Ultrasonic Motors

**DOI:** 10.3390/mi17040400

**Published:** 2026-03-25

**Authors:** Zhiling Liu, Qiufeng Yan, Qingyu Liu

**Affiliations:** 1 Graduate School of Education, Joongbu University, Goyang 10279, Republic of Korea; liuzl044926@163.com; 2 School of Electrical Engineering and Automation, Nantong University, Nantong 226019, China; yanqf@nuaa.edu.cn; 3 School Key Laboratory of Mechanics and Control for Aerospace Structures, Nanjing University of Aeronautics and Astronautics, Nanjing 210016, China; 4 School of Engineering, Nanfang College, Guangzhou, Guangzhou 510970, China

**Keywords:** LUSMs, traveling wave, standing wave, progress in application, development trend

## Abstract

Linear ultrasonic motors (LUSMs) occupy an important position in the field of high-precision actuation due to their advantages of simple structure, high control accuracy and direct linear motion generation. This review first classifies LUSMs according to wave modes into traveling wave linear ultrasonic motors (TWLUSMs) and standing wave linear ultrasonic motors (SWLUSMs). Among them, TWLUSMs include the straight beam type and the annular beam type, while SWLUSMs consist of the single-foot type and the multi-foot type. In addition, the working principles of TWLUSMs and SWLUSMs are elaborated. The structural characteristics and performance parameters of different types of ultrasonic motors (USMs) are sorted out, and the analysis shows that SWLUSMs are significantly superior to TWLUSMs in terms of output speed and output force. This review summarizes the application status of LUSMs in fields such as biomedicine, deep-sea exploration, aerospace and precision manufacturing, and finally outlines the development trends of LUSMs from the aspects of miniaturization and lightweighting, extreme environment adaptability and intelligent upgrade. This review provides a comprehensive reference for the structural design, performance improvement and application expansion of LUSMs.

## 1. Introduction

USMs [1,2,3] are a new type of motor that achieves actuation by utilizing ultrasonic vibrations. Owing to their advantages of compact structure, simple miniaturization and high positional resolution, they have gradually replaced traditional electromagnetic motors in the field of high-precision applications. In 1942, Williams et al. [4] invented the first USM. Although this USM failed to achieve practical applications, it fully demonstrated the operating principles of USMs. In 1985, Sashida [5] proposed the traveling wave USM and elaborated on its operating principles, and Canon applied this USM to the autofocus system of cameras. Since then, USMs have entered the stage of practical application.

**Figure 1:**
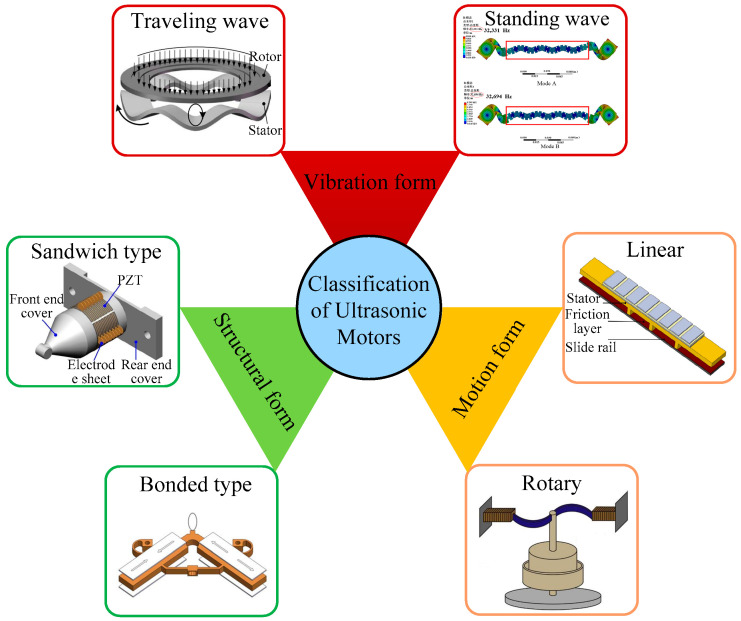
Classification of USMs.

As shown in Figure 1, USMs can be classified into traveling wave USMs [6] and standing wave USMs [7] according to their vibration modes. Based on their motion forms, USMs can be classified into LUSMs [8] and rotary USMs [3]. USMs can be classified into sandwiched ultrasonic motors [9] and bonded ultrasonic motors [10] in terms of their structural characteristics. Among these classification methods, categorization based on the motion form of the mover is the most prevalent. The LUSM converts the micro-vibration of the stator into the linear motion of the mover through the frictional interaction between the stator and the mover.

Compared with rotary USMs, LUSMs have the advantages of simple structure, high control accuracy and direct linear motion generation. Consequently, they have attracted extensive attention from scholars [11,12,13] and have been widely applied in fields such as biomedicine [14], deep-sea exploration [15], aerospace [16], and precision manufacturing [17]. LUSMs can be classified into TWLUSMs [18] and SWLUSMs [19] according to their wave modes. Among them, TWLUSMs can be divided into straight beam type [20] and annular beam type [9], while SWLUSMs can be categorized into single-foot type [21] and multi-foot type [22].

At present, some scholars have reviewed the research status of LUSMs. Yao et al. [23] mainly elaborated on the structural design, theoretical models and application progress of LUSMs, but they did not classify and introduce the research progress of LUSMs or clarify the performance characteristics of various types of LUSMs. Shalini et al. [24] introduced the working principles, the characteristics of different types of LUSMs and their research progress, yet they failed to conduct a classified discussion on the research progress of different types of LUSMs.

This review classifies LUSMs into TWLUSMs and SWLUSMs, elaborates on the research progress on these two types, respectively, and compares their respective performance characteristics. Finally, it summarizes the application status of LUSMs and points out the development trends of LUSMs.

## 2. Principle of LUSMs

**Figure 2:**
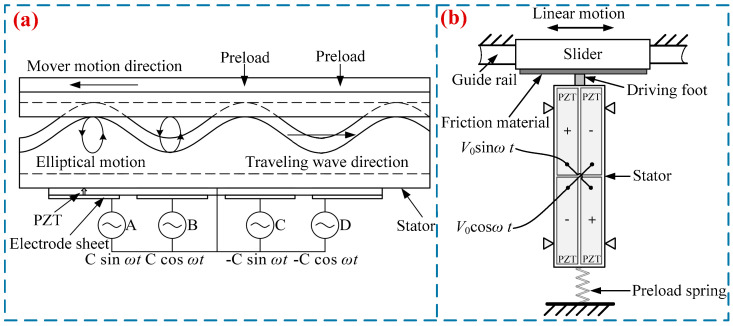
Working principle of LUSMs: (**a**) traveling wave type, (**b**) standing wave type.

An LUSM is a driving device that converts electrical energy into the ultrasonic vibrations of an elastomer and then transforms such vibrations into the linear motion of a mover via friction-driven transmission. Figure 2a shows the working principle of the TWLUSMs. First, a bending traveling wave is excited in the stator, which causes the particles on its surface to form driving elliptical motion trajectories. Then, through the frictional coupling between the stator and the guide rail, the micro-vibration energy of the particles on the stator surface is converted into the mechanical energy of the guide rail, thereby driving the guide rail to produce linear motion. Figure 2b shows the working principle of the SWLUSMs. When voltage signals with the same frequency but a 90° phase difference are applied to piezoelectric ceramic plates with different polarization directions on the stator. The first-order in-plane longitudinal vibration and second-order bending vibration modes of the stator will be excited, thereby forming an elliptical motion at the driving feet of the stator. Under the action of preload, a frictional effect is generated to achieve the driving of the slider.

## 3. Current Status of Research on LUSMs

### 3.1. TWLUSMs

In 1983, Sanshida proposed the earliest TWLUSM, which was structurally categorized into the straight beam type and the annular beam type [25]. The straight beam TWLUSM [20] employs two Langevin transducers arranged at both ends of the straight beam, one for excitation and the other for vibration absorption. When the excitation transducer is energized, a traveling wave propagating forward is generated on the straight beam. Reversing the positions of the excitation transducer and the vibration absorption transducer enables the traveling wave to reverse direction, thereby driving the slider to move reversely. In contrast, the annular beam TWLUSM [9] adopts two groups of piezoelectric ceramics mounted at the bottom of the annular beam and utilizes the superposition of two sets of standing waves to form a traveling wave to drive the slider.

#### 3.1.1. Straight Beam Type TWLUSM

**Figure 3:**
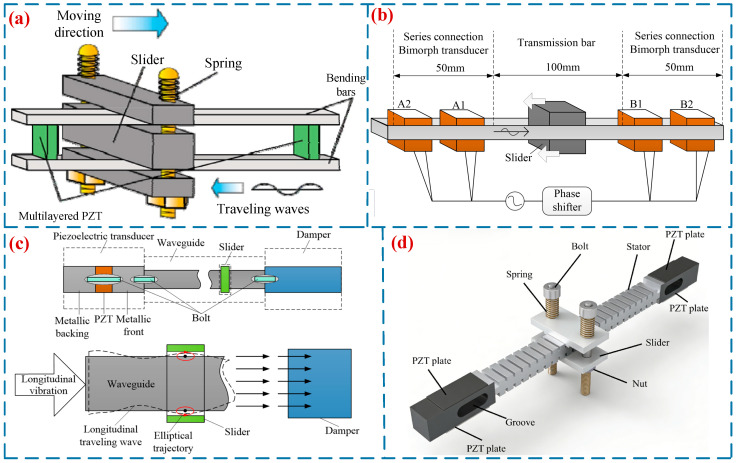
TWLUSMs: (**a**) a miniature TWLUSM with a rod structure proposed by Kondo et al. [27], Copyright (2010) Elsevier. (**b**) a miniature TWLUSM with a straight beam structure proposed by Kondo et al. [28], (**c**) a rod-type TWLUSM proposed by Wang et al. [29], (**d**) a TWLUSM proposed by Wen et al. [20].

Ting et al. [26] proposed a TWLUSM with a broken-line structure, which is integrally shaped into a serpentine, zigzag configuration mimicking snake locomotion. When the driving voltage is 140 V and the driving frequency is 26.58 kHz, the motor achieves a maximum output speed of 36.14 mm/s and a maximum force of 2.76 N. Kondo et al. [27] proposed a miniature TWLUSM with a rod structure (Figure 3a). This USM excites the ceramic plates to induce bending vibration of the rod structure, while generating a traveling wave on the rod structure to achieve motion output. Experimental results demonstrate that the slider reaches an output speed of 12 mm/s with a maximum output force of 0.075 N. Subsequently, Kondo et al. [28] proposed another miniature TWLUSM with a straight beam structure (Figure 3b). This USM employs two series of bimorph vibrators bonded to the two ends of the straight beam, respectively, which excite two standing waves that superimpose on the straight beam to form a traveling wave. At a driving frequency of 66 kHz, this USM achieves a maximum output speed of 15 mm/s and a maximum output force of 0.5 N. Wang et al. [29] proposed a rod-type TWLUSM (Figure 3c), with an output speed of 55 mm/s. Wen et al. [20] proposed a TWLUSM (Figure 3d), teeth were added to the beam to amplify the transverse displacement, thereby improving the mechanical output performance. A prototype with dimensions of 10 × 10 × 160 mm^3^ was fabricated and tested. At a driving voltage of 300 V and a driving frequency of 32.7 kHz, the prototype achieved an output speed of 53.7 mm/s and an output force of 0.83 N. Table 1 shows the performance parameters of straight-beam-type TWLUSMs.

#### 3.1.2. Annular Beam Type TWLUSM

**Figure 4:**
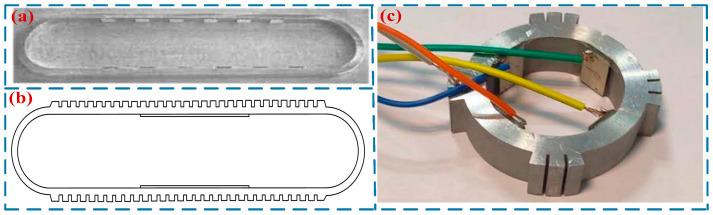
TWLUSMs: (**a**) a circular TWLUSM proposed by He et al. [31], Copyright (2003) China Electronics Technology Group Corporation. (**b**) an annular TWLUSM proposed by Liu et al. [33], (**c**) an annular TWLUSM with incomplete teeth proposed by Yang et al. [34]. Copyright (2022) China Electronics Technology Group Corporation.

It is difficult to achieve impedance matching between the two vibrators in a straight-beam-type TWLUSM, and severe wave reflection problems exist on the straight beam. To mitigate the issue of traveling wave reflection, Seemann [30] developed an annular-beam-type TWLUSM in 1996. Two groups of piezoelectric ceramics were mounted at the bottom of the annular beam, and the superposition of two sets of standing waves was utilized to form a traveling wave to drive the slider. He et al. conducted in-depth research on annular-beam-type TWLUSMs, proposing a circular traveling wave LUSM [31] (Figure 4a) and a square-ring traveling wave LUSM [32], respectively. Meanwhile, they performed modal analysis and experimental research on both motors. Liu et al. [33] designed an annular TWLUSM (Figure 4b). Several teeth were added to the upper and lower straight beam segments of the stator to amplify the amplitude, and when the driving voltage is 300 V, the motor achieved a maximum output speed of 15 mm/s. Yang et al. [34] proposed a novel annular TWLUSM with incomplete teeth (Figure 4c). At a driving voltage of 240 V and a driving frequency of 30.459 kHz. This USM achieves a speed of 102 mm/s and a maximum output force of 90 mN. Annular-beam-type TWLUSMs can address the traveling wave reflection issue associated with straight-beam-type TWLUSMs. However, the entire substrate vibrates during operation, which results in high system heat generation and low efficiency. Meanwhile, this type of USM also features a large volume and complex structure. Consequently, relatively few researchers have conducted studies on this type of USM. Table 2 shows the performance parameters of annular-beam-type TWLUSMs.

In summary, the traveling wave mode has been widely adopted in rotary USMs [9,35,36]. However, TWLUSMs suffer from low output driving force, low efficiency, and large volume. It can thus be concluded that the traveling wave mode is not well suited for LUSMs.

### 3.2. SWLUSMs

TWLUSMs [20] have problems such as large volume, high power consumption, and low efficiency. Researchers have gradually turned their attention to SWLUSMs [37]. Compared with TWLUSMs, SWLUSMs have the advantages of simple structure, flexible design, and high output efficiency. SWLUSMs achieve actuation by exciting a standing wave in the stator, and the motion trajectory of their driving feet tends to be an approximate oblique line [38]. Since Sashida [39] proposed the SWLUSM in 1982, the SWLUSM has attracted extensive attention from numerous researchers. SWLUSMs can be classified into single-footed SWLUSMs [40] and multi-footed SWLUSMs [41] according to the number of driving feet.

#### 3.2.1. Single-Footed SWLUSMs

**Figure 5:**
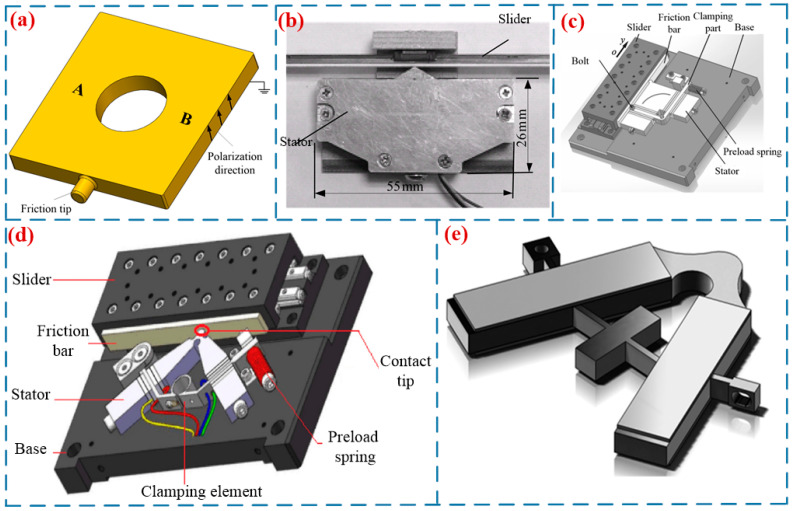
Single-footed SWLUSMs: (**a**) an SWLUSM with a novel structure proposed by Chen et al. [19], (**b**) an SWLUSM proposed by Shi et al. [43], Copyright (2015) Hans. (**c**) a high-thrust LUSM with an L-shaped stator proposed by Zhang et al. [44], Copyright (2018) Xi’an Jiaotong University. (**d**) a novel V-shaped LUSM proposed by Zhou et al. [10], Copyright (2018) Guizhou Institute of Technology. (**e**) a V-shaped LUSM with a hinged support proposed by Yang et al. [46]. Copyright (2017) China Association for Science and Technology.

In 1990, Endo et al. [42] proposed a single-footed SWLUSM, which achieved a maximum force of 2 N and a maximum speed of 20 mm/s. Chen et al. [19] proposed an SWLUSM with a novel structure (in Figure 5a). By exciting two regions of the vibrator, the driving feet generate two orthogonal approximate linear trajectories, thereby driving the mover to perform reciprocating linear motion. This motor achieves a maximum output speed of 310 mm/s, a maximum output force of 2.35 N, and an efficiency of 14.5%. Shi et al. [43] proposed an SWLUSM with composite modal vibration (see Figure 5b). An isosceles triangular structure was adopted as the driving foot to actuate the slider, and under a preload of 24 N, the motor achieved a speed of 100 mm/s and a maximum force of 2.5 N. Zhang et al. [44] proposed a high-thrust LUSM with an L-shaped stator (see Figure 5c). The stator is composed of two mutually perpendicular rectangular plate vibrators, and one of the vibrators is installed parallel to the slider, which renders the motor structure more compact. The motor achieves a maximum no-load speed of 384 mm/s and an output force of 90 N. Liu et al. [45] proposed a novel I-shaped LUSM. Composed of two rectangular piezoelectric vibrators, the motor has the two vibrators mounted parallel to the slider. This motor weighs a mere 18.2 g, with a maximum output speed of 364 mm/s and an output force of 4.9 N, respectively. The V-shaped linear motor is a typical single-footed SWLUSM (in Figure 5d). Composed of a V-shaped piezoelectric transducer (stator), a mover, a clamping device, and a base [10], it boasts such merits as high resolution, fast response, high output velocity, and large output force, thus attracting extensive attention from researchers. Yang et al. [46] designed a V-shaped LUSM with a hinged support at one end (see Figure 5e), which simplified the structure of the ultrasonic motor. At an included angle of 90°, a driving voltage of 300 V and a preload of 40 N, the motor achieved a 66.8% increase in no-load velocity, a 55% improvement in load capacity, a maximum no-load speed of 784 mm/s and a maximum output force of 14 N. Huang et al. [47] proposed a low-voltage V-shaped LUSM. At a driving frequency of 37 kHz and a driving voltage of 50 V, the motor achieves a maximum output force of 25.8 N and a maximum no-load speed of 1.221 m/s. In addition, Su et al. [48], Delibas et al. [21], and other researchers have also conducted studies on single-footed standing-wave linear ultrasonic motors. Table 3 shows the performance parameters of the single-footed SWLUSMs.

#### 3.2.2. Multi-Footed SWLUSMs

**Figure 6:**
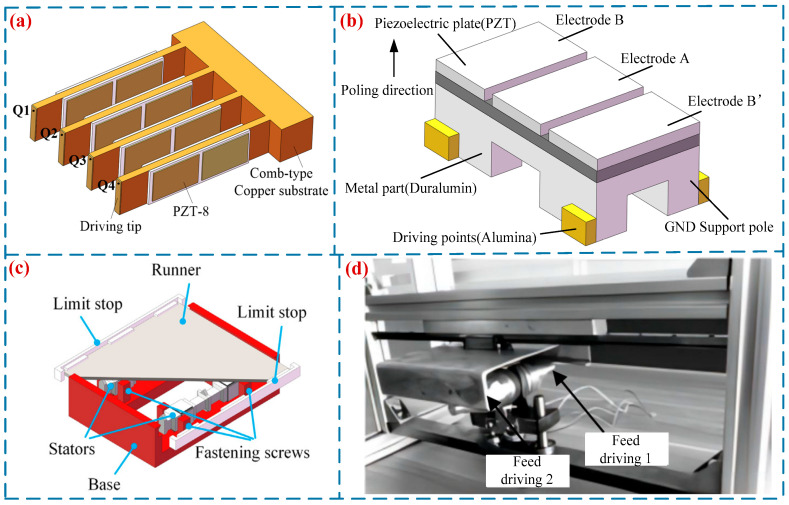
Multi-footed SWLUSMs: (**a**) a multi-footed LUSM proposed by Zhu et al. [50], (**b**) a quadruped SWLUSM proposed by Tanoue et al. [52], (**c**) a tripod SWLUSM proposed by Li et al. [53], Copyright (2021) Elsevier. (**d**) a cylindrical bipedal LUSM proposed by Fu et al. [54]. Copyright (2021) China Electronics Technology Group Corporation.

To improve the working efficiency of LUSMs, researchers have designed LUSMs driven by multiple driving feet. In 1991, Ohnishi [49] proposed a π-type LUSM, which featured a dual-driving-foot configuration. This design could significantly enhance the utilization rate of the stator during vibration and improve the output performance of the USM. Zhu et al. [50] proposed a novel LUSM suitable for rapid ultra-precision positioning (in Figure 6a). This ultrasonic motor is composed of a comb-shaped structure, eight piezoelectric ceramic plates and four ceramic driving feet. By altering the driving signals, the motor can operate in both the rapid-driving mode and the precision positioning mode. In the rapid-driving mode, when the driving voltage is 200 V, the motor attains a maximum no-load speed of 181.2 mm/s and a maximum output force of 1.7 N. In the precision positioning mode, it achieves a positioning accuracy of 0.08 μm. Jian et al. [51] proposed a II-type dual-foot LUSM, whose maximum output speed and maximum output force reached 273 mm/s and 110 N, respectively, thus significantly improving the working efficiency of the USM. Tanoue et al. [52] proposed a quadruped SWLUSM (in Figure 6b). This motor achieved a maximum output speed of 148 mm/s and a maximum output force of 0.294 N when operated at a driving voltage of 160 V and a driving frequency of 84.8 kHz. Li et al. [53] developed a tripod SWLUSM (in Figure 6c). Each stator of this motor is equipped with three driving feet at its middle and two ends. During operation, these driving feet simultaneously perform elliptical trajectory motions of the same pattern to drive the slider, thereby enhancing the output performance of the motor. When driven at a voltage of 65 V and a frequency of 34.72 kHz, the motor achieves a maximum no-load speed of 117.22 mm/s. Fu et al. [54] developed a cylindrical bipedal LUSM (in Figure 6d). The left and right parts of the motor are fastened by studs, and the micro-vibration of the driving feet at both ends of the vibrator is utilized to achieve bipedal driving. The tapered horn structures on both sides of the motor amplify the amplitude of the driving feet. Subsequent optimization of the motor’s structural parameters enabled it to reach a maximum speed of 320 mm/s and a maximum force of 11 N.

In addition, first longitudinal second bending mode (L1B2) USMs [55,56] are also one of the key research objects in the field of USMs. Wischnewski et al. [57], Zhu et al. [58], Lee et al. [22], Wang et al. [37] and other research teams have also conducted investigations on multi-footed SWLUSMs. Table 4 presents the performance parameters of multi-footed SWLUSMs.

### 3.3. Comparison of TWLUSMs and SWLUSMs

**Figure 7:**
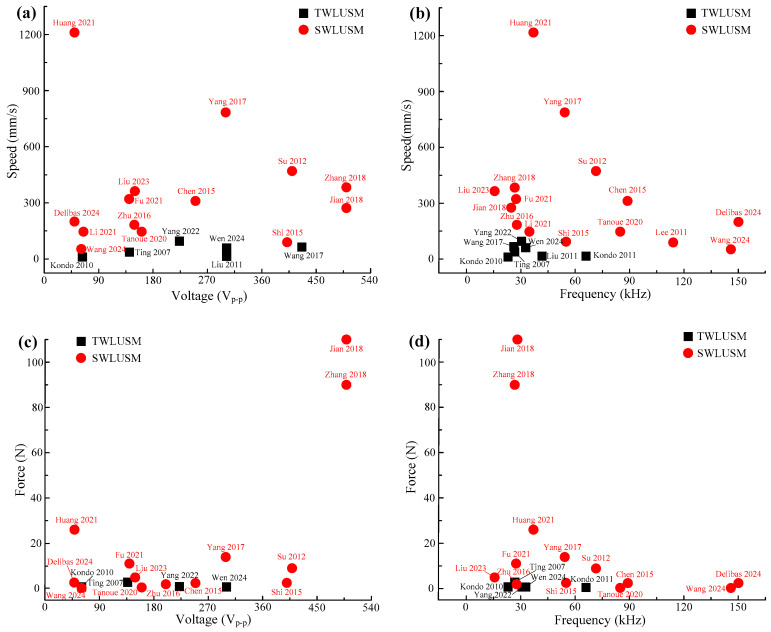
Maximum speed and force for TWLUSMs and SWLUSMs [19,21,22,26,27,28,29,33,34,37,43,44,45,46,47,48,50,51,52,53,54]: (**a**,**b**) speed versus voltages and frequencies, (**c**,**d**) force versus voltages and frequencies.

Figure 7 shows the output speed and output force characteristics of TWLUSMs and SWLUSMs. TWLUSMs exhibit inferior output performance, with their output speed generally below 100 mm/s and output force typically less than 1 N, peaking at merely 2.76 N. SWLUSMs demonstrate superior output performance, with their output speed usually exceeding 100 mm/s and even capable of surpassing 1000 mm/s, while their output force is typically above 2 N and can reach a maximum of over 100 N. The reasons for the inferior output performance of TWLUSMs are as follows:

Severe traveling wave reflection occurs in the straight beam TWLUSMs, which leads to unstable operation or low efficiency of the motors. Although the annular beam TWLUSMs can address the issue of traveling wave reflection, its excessively large ring-beam structure results in low operational efficiency of the motor. In contrast, the SWLUSM achieves actuation by exciting a standing wave within the stator. This design can effectively overcome the issues of poor output performance and low efficiency, thereby significantly enhancing the operational efficiency and performance of the motor.

In addition, based on the summarized research progress of TWLUSMs and SWLUSMs above, TWLUSMs have garnered relatively little attention, and traveling waves are not suitable for driving LUSMs. For the field of linear actuation, researchers mainly achieve linear motion via SWLUSMs and have successively proposed the L-shaped LUSM [44], the I-shaped LUSM [45], the V-shaped LUSM [46], and other configurations.

## 4. Research Progress on Friction Interface of USMs

USMs transmit power and drive loads via the interfacial friction between the friction layer and the stator, and friction materials and structures exert a significant impact on the performance and service life of USMs [59,60].

### 4.1. Friction Materials

The motion mechanism of USMs demands that friction materials have suitable stiffness, friction coefficient and wear resistance. Therefore, numerous researchers have conducted studies on the friction materials used in USMs. In 1998, Rehbein et al. [61] adopted composites of polytetrafluoroethylene (PTFE) and polyimide (PI) as friction materials for USMs, which effectively prolonged the service life of the motors but failed to meet the requirements in terms of output performance. Qu et al. [62] prepared novel friction materials by adding various modifiers and reinforcing fibers into polymer matrices such as polybenzoate and PTFE, which significantly improved the friction coefficient and wear resistance of the materials as well as the output performance of USMs. Li et al. [63] developed a novel PI friction material. Compared with conventional PTFE, this new-type PI friction material exhibited a remarkably enhanced efficiency. Shang et al. [64] investigated the effects of fluorite on the physical, mechanical and tribological properties of friction composites. Moreover, research shows that fluorite can inhibit the degradation of phenolic resin, improve the thermal stability of friction materials, and effectively reduce the wear rate. Qu et al. [65] reduced the wear rate of friction materials, diminished noise and ineffective vibrations, and significantly enhanced the stability and service life of USMs by incorporating friction modifiers with excellent frictional and anti-wear properties.

### 4.2. Friction Layer Interface Structure

The friction layer interface structure exerts a significant impact on the energy conversion efficiency, friction loss and service life of USMs. Li et al. [66] fabricated surface textures with diverse distributions on the stator surfaces of USMs and investigated the interfacial friction state and friction loss between the friction layer and the stator. The results indicate that surface texturing can effectively reduce the wear debris generated by the friction layer material during motor operation, which accumulates on the stator surface to form a stator transfer film, thus prolonging the service life of the friction layer. Hua et al. [67] fabricated high-precision micro-groove arrays at the friction layer interface of USMs and experimentally verified that surface texturing can significantly increase the friction coefficient of contact surfaces and reduce the wear rate. Li et al. [66] pointed out that surface texturing transforms the wear mechanism of friction materials from adhesive wear into fatigue wear caused by alternating stress. Since fatigue wear is relatively stable, it can prolong the service life of USMs.

## 5. Applications of LUSMs

LUSMs boast advantages such as simple structure, high control accuracy and direct linear motion generation, thus being widely applied in fields including biomedicine, deep-sea exploration, aerospace, and precision manufacturing.

### 5.1. Biomedicine

Compared with electromagnetic motors, USMs have significant advantages in miniaturization, low speed and high torque, no electromagnetic interference, and fast response, making them particularly suitable for fields such as gastrointestinal endoscopy and minimally invasive surgery.

**Figure 8:**
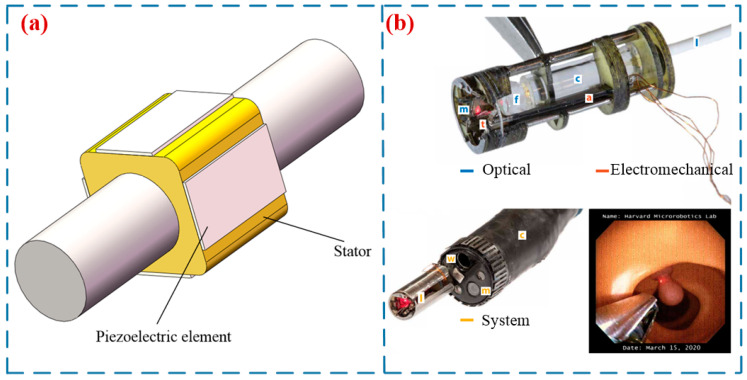
Applications of LUSMs in biomedicine: (**a**) a miniature rotary–linear USM proposed by Mashimo et al. [68], (**b**) an LUSM designed by York et al. [69] for minimally invasive surgery, Copyright (2021) Science.

In 2008, Mashimo et al. [68] proposed a miniature rotary–linear USM for intravascular diagnosis and surgery (in Figure 8a). The stator of this motor is a cube, and the rotor is a cylinder. At a resonant frequency of 306 kHz, its speed and force can reach 50 mm/s and 0.01 mN. York et al. [69] designed an ultrasound-motor-based laser guidance device for minimally invasive surgery. This device has a diameter of 6 mm and a length of 16 mm, and can focus and control laser beams transmitted through optical fibers over a wide range. Chen et al. [70] proposed a miniature hollow LUSM (in Figure 8b), which enables precise optical zooming within the endoscope system. Shen et al. [71] proposed a novel drug delivery method that integrates piezoelectric actuation and ultrasonic technology for the biopsy channel of an endoscope. By inserting a bidirectional piezoelectric actuator into an endoscope equipped with an ultrasonic transducer, the distance between the transducer and the lesion can be adjusted to enhance the drug delivery rate. In addition, Lan et al. [14] and Krieger et al. [72] have applied LUSMs in the fields of surgery and nuclear magnetic resonance, respectively.

### 5.2. Deep-Sea Exploration

With the growing development of human ocean exploration, increasingly stringent requirements have been put forward for actuators capable of continuous and stable operation in the deep sea. The pressure in the deep sea can reach up to 110 MPa, under which conventional electromagnetic motors generally fail to work stably. USMs feature a simple structure, they can achieve underwater motion only by sealing the electrodes and piezoelectric materials, thus boasting broad application prospects in underwater environments, even in the deep sea. In recent years, they have attracted extensive attention from researchers.

**Figure 9:**
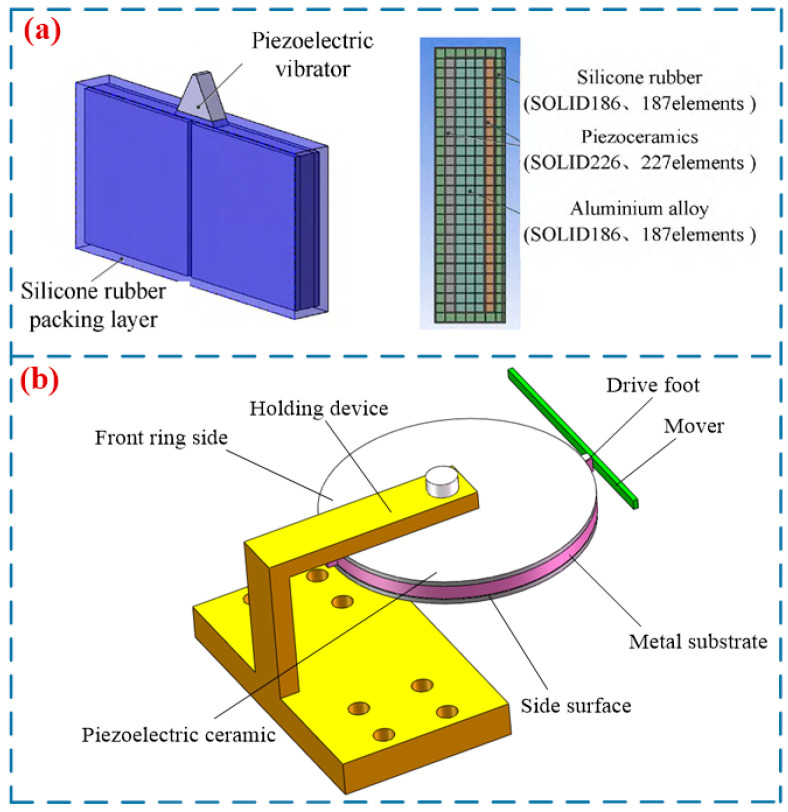
Applications of LUSMs in deep-sea exploration: (**a**) a novel resonant LUSM proposed by He et al. [73], Copyright (2018) IEEE. (**b**) an LUSM proposed by Zhang et al. [75].

In 2018, Jiang et al. [15] developed a three-degree-of-freedom piezoelectric actuator for deep-sea robotic arms. At a driving frequency of 37.4 kHz and a driving voltage of 310 V, the output torque of the actuator reached 0.07 N.m. He et al. [73] proposed a novel resonant LUSM for deep-sea applications, the structure of which is illustrated in Figure 9a. At a water pressure of 8 MPa, a driving frequency of 72 kHz and a driving voltage of 200 V, the motor achieved a moving speed of 214 mm/s. Yu et al. [74] proposed an underwater robotic finger joint driven by a patch-type piezoelectric actuator. The maximum underwater motion speed of the finger joint reached 689 deg/s, and its maximum torque was 13.18 mN·m. This design provides a novel approach for the joint actuation of underwater robots. Zhang et al. [75] proposed an LUSM capable of achieving stable and efficient linear actuation in deep-sea environments (in Figure 9b), which serves as an actuator for small underwater robotic arms. This motor achieves a maximum driving speed of 376 mm/s and a maximum load capacity of 2.5 N on land. Under an underwater pressure of 8 MPa, its maximum driving speed reaches 215 mm/s.

### 5.3. Aerospace

**Figure 10:**
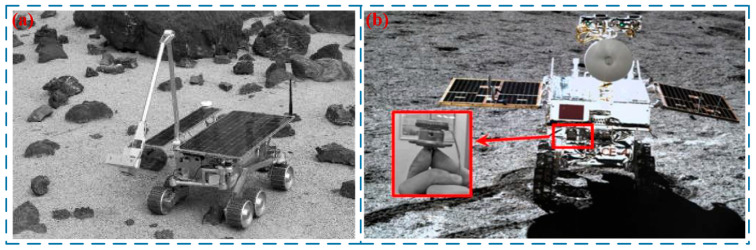
Applications of LUSMs in aerospace: (**a**) a three-ring TWUSM proposed by Weisbin et al. [77], Copyright (1999) ISAIRAS ’99. (**b**) USMs proposed by Wu et al. [78], Copyright (2021) Engineered Science.

Compared with electromagnetic motors, USMs can avoid the adverse effects of harsh environments such as high vacuum, extreme temperatures and high radiation. In addition, USMs feature a compact structure, high flexibility and ease of miniaturization, which can help reduce the volume and mass of aerospace detectors. USMs have been applied in the aerospace industry of the United States. In 1999, Das et al. [76] proposed a USM for Mars micro-landers. Weisbin et al. [77] from the National Aeronautics and Space Administration (NASA) developed a three-ring TWUSM, which was applied to micromanipulation on space robots and exhibited excellent stability and reliability. Wu et al. [78] applied USMs to the Chang’e-3 and Chang’e-4 lunar probes (in Figure 10a). The current response time of these motors ranges from 1 to 30 ms, and the shutdown response time is 0.1 to 1 ms, with their weight merely one-tenth that of conventional motors. Liu et al. [79] designed a small morphing wing driven by distributed USMs based on a V-shaped LUSM, which can be used for the intelligent morphing actuation and control of wing profiles during low-speed flight. Li et al. [16] proposed a USM applicable to the actuation of solar wings for micro–nano satellites (in Figure 10b). This motor can meet the requirements of miniaturization and lightweight design, enhance energy supply capacity simultaneously, and achieve an energy saving rate of 23.8% compared with conventional actuation methods.

### 5.4. Precision Manufacturing

**Figure 11:**
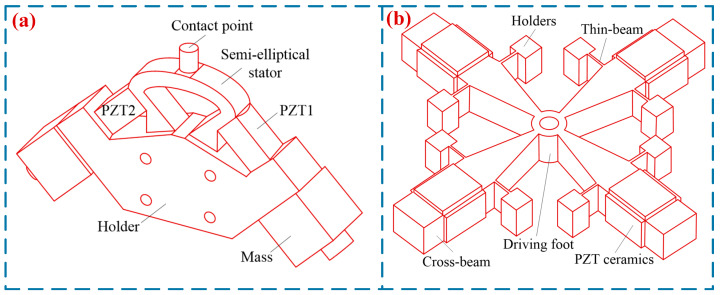
Applications of LUSMs in precision manufacturing: (**a**) a novel precision USM proposed by Ho et al. [80], Copyright (2016) Elsevier. (**b**) a piezoelectric actuator with three degrees of freedom proposed by Wang et al. [82].

LUSMs feature fast response, high precision, and high resolution, enabling micro-displacement adjustment and precise motion control, and thus are widely used in the field of precision manufacturing. Ho et al. [80] proposed a novel precision USM (in Figure 11a). This motor is capable of achieving high-precision positioning, with a displacement resolution of 6 nm when operated at a driving frequency of 100 Hz. Li et al. [81] designed and fabricated a linear motion platform, which can achieve low-speed motion with the error controlled within 3%. Wang et al. [82] designed a piezoelectric actuator with three degrees of freedom (in Figure 11b). At a driving voltage of 200 V and a rotor mass of 4 g, the output speeds of the *x*, *y*, and *z* axes reached 363 rpm, 342 rpm, and 171 rpm, respectively. Zhou et al. [17] significantly improved the positioning accuracy of USMs via a flexible shaft. Experimental results demonstrate that the USM with a flexible shaft achieves a minimum positioning error of 1.79 μrad, with a positioning response time of less than 1 s.

In addition, LUSMs have also found extensive applications in fields such as optical engineering [83], walking assistive systems [84] and robotics [85].

## 6. Development Trends of LUSMs

### 6.1. Miniaturization and Lightweighting

With the trend toward high precision in manufacturing industries, there is an urgent demand for compact, direct-drive miniaturized LUSMs in fields such as semiconductor manufacturing, advanced military equipment, aerospace engineering, and biomedical engineering. Over the past decade, miniaturization of SWLUSMs based on thin plate-shaped and frame-shaped stator structures has emerged as a primary research direction. Shunsuke et al. [86] developed the smallest LUSM, with a volume of merely 4.5 × 4.5 × 1 mm^3^, an output speed of 140 mm/s, and an output force of 12.9 mN. However, miniaturization is not the sole objective in the development of ultrasonic motors. It is imperative to ensure that ultrasonic motors possess favorable output performance, stability, and wear resistance while achieving miniaturization and lightweighting. In the future, millimeter-scale or even micron-scale LUSMs can be developed by leveraging Micro-Electro-Mechanical Systems (MEMS) technology, ultra-thin piezoelectric materials, and lightweight composite materials. Meanwhile, the weight of the motors can be controlled to below 1 g, while ensuring that their output performance meets the requirements of driving applications.

### 6.2. Extreme Environment Adaptability

When USMs are applied in deep-sea and space exploration, they operate under extremely harsh working environments. Conventional USMs tend to exhibit unstable performance under high pressure, extreme temperatures and radiation conditions, which will restrict their practical applications. Preliminary applications of USMs have been achieved under the conditions of 8 MPa pressure in deep-sea environments and high-vacuum environments [75]. However, for extreme environments such as high hydrostatic pressure (110 MPa in deep-sea scenarios), extreme temperatures and intense radiation (in space environments), the reliability of USMs still needs to be further improved. In the future, the range of applications of USMs in fields like deep-sea exploration and space exploration will be expanded through the application of polymer composite materials, development of piezoelectric materials resistant to extreme environments, and structural design with high-temperature stability.

### 6.3. Intelligent Upgrade of the Control and Drive Section

Currently, a single control and drive function can hardly meet the requirements of complex equipment. In the future, the integrated design of LUSMs with components such as sensors, controllers and reducers will be promoted to form an integrated “drive-sensing-control” module. Furthermore, integrating AI algorithms into the control and drive sections of LUSMs enables real-time monitoring of their operating status; collects parameters such as rotational speed, current, and temperature of LUSMs; and uses AI algorithms to determine the working state of LUSMs, adjusting the control strategy in a timely manner to achieve precise control.

## 7. Conclusions

Based on wave forms, this review classifies LUSMs, elaborates on their operating principles, summarizes the current research status of LUSMs, and introduces their application fields as well as future development trends. The specific conclusions of this review are as follows:

(1) LUSMs are classified into TWLUSMs and SWLUSMs according to their wave forms, where TWLUSMs includes the straight beam type and the annular beam type, while SWLUSMs consists of the single-footed type and the multi-footed type. The operating principles of TWLUSMs and SWLUSMs are elaborated, respectively.
(2) The research progress of TWLUSMs and SWLUSMs is elaborated by category, and a comparison of their output performance indicates that TWLUSMs yield relatively low output speed and force with fewer relevant research findings, while SWLUSMs demonstrate distinct advantages in output speed and force, thus garnering substantial attention from scholars.
(3) At present, LUSMs have achieved practical breakthroughs in numerous fields, particularly being widely applied in areas such as biomedicine, deep-sea exploration, aerospace, and precision manufacturing.
(4) Miniaturization and lightweighting, enhanced adaptability to extreme environments and intelligent upgrade will represent the future development trends of LUSMs.

## Figures and Tables

**Table 1:** Parameters of the straight-beam-type TWLUSM.

Year	First Author and Reference	*V*_p_ (L × H × W) (mm^3^)	Voltage (V)	Frequency (kHz)	Speed (mm/s)	Force (N)
2007	Ting [26]	/	140	26.58	36.14	2.76
2010	Kondo [27]	/	60	23	12	0.075
2011	Kondo [28]	100 × 4 × 8	/	66	15	0.5
2017	Wang [29]	/	420	25.3	55	/
2024	Wen [20]	160 × 10 × 10	300	32.7	53.7	0.83

“/” indicates that the corresponding data were not reported in the referenced literature.

**Table 2:** Parameters of the annular-beam-type TWLUSM.

Year	First Author and Reference	*V*_p_ (L × H × W) (mm^3^)	Voltage (V)	Frequency (kHz)	Speed (mm/s)	Force (N)
2011	Liu [33]	/	300	41.68	15	/
2022	Yang [34]	12 × 6 × 1	240	30.459	102	0.09

“/” indicates that the corresponding data were not reported in the referenced literature.

**Table 3:** Parameters of single-footed SWLUSMs.

Year	First Author and Reference	*V*_p_ (L × H × W) (mm^3^)	Voltage (V)	Frequency (kHz)	Speed (mm/s)	Force (N)
2015	Chen [19]	15 × 15 × 2	250	89	310	2.35
2015	Shi [43]	55 × 26 × 14	400	55	100	2.5
2018	Zhang [44]	80 × 70 × 11	500	26.6	384	90
2023	Liu [45]	39.8 × 17.6 × 6	150	15.5	364	4.9
2017	Yang [46]	/	300	54.2	784	14
2021	Huang [47]	20 × 20 × 0.5	50	37	1221	25.8
2012	Su [48]	33 × 20 × 3	400	71.4	470	9
2024	Delibas [21]	7 × 7 × 2	50	150	200	2.5

“/” indicates that the corresponding data were not reported in the referenced literature.

**Table 4:** Parameters of multi-footed SWLUSMs.

Year	First Author and Reference	*V*_p_ (L × H × W) (mm^3^)	Voltage (V)	Frequency (kHz)	Speed (mm/s)	Force (N)
2016	Zhu [50]	33 × 32.6 × 7	150/200	27.9	181.2	1.7
2018	Jian [51]	/	500	24.8/28.0	273	110
2020	Tanoue [52]	20 × 12 × 6.6	160	84.8	148	0.294
2021	Li [53]	74 × 10 × 10	65	34.72	117.22	/
2021	Fu [54]	/	140	27.4	320	11
2011	Lee [22]	9 × 8 × 1	/	114	88	/
2024	Wang [37]	8 × 1 × 0.6	60	145.8	53.3	0.043

“/” indicates that the corresponding data were not reported in the referenced literature.

## Data Availability

The original contributions presented in this study are included in the article. Further inquiries can be directed to the corresponding author.
